# Development, reliability and factor analysis of a self-administered questionnaire which originates from the World Health Organization's Composite International Diagnostic Interview – Short Form (CIDI-SF) for assessing mental disorders

**DOI:** 10.1186/1745-0179-4-8

**Published:** 2008-04-10

**Authors:** Antonella Gigantesco, Pierluigi Morosini

**Affiliations:** 1 Mental Health Unit, National Centre of Epidemiology, Surveillance and Health Promotion, Italian National Institute of Health, Rome, Italy

## Abstract

**Background:**

The Composite International Diagnostic Interview – Short Form consists of short form scales for evaluating psychiatric disorders. Also for this version training of the interviewer is required. Moreover, the confidentiality could be not adequately protected.

This study focuses on the preliminary validation of a brief self-completed questionnaire which originates from the CIDI-SF.

**Sampling and Methods:**

A preliminary version was assessed for content and face validity. An intermediate version was evaluated for test-retest reliability. The final version of the questionnaire was evaluated for factor exploratory analysis, and internal consistency.

**Results:**

After the modifications by the focus groups, the questionnaire included 29 initial probe questions and 56 secondary questions. The test retest reliability weighted Kappas were acceptable to excellent for the vast majority of questions. Factor analysis revealed six factors explaining 53.6% of total variance. Cronbach's alpha was 0.89 for the questionnaire and 0.89, 0.67, 0.71, 0.71, 0.49, and 0.67, for the six factors respectively.

**Conclusion:**

The questionnaire has satisfactory reliability, and internal consistency, and might be efficient for using in community research and clinical practice. In the future, the questionnaire could be further validated (i.e., concurrent validity, discriminant validity).

## 1. Introduction

The Composite International Diagnostic Interview (CIDI) was developed by the World Health Organisation (WHO) for assessing mental disorders according to the definitions of ICD-10 and DSM-IV [1]. It is intended for use in general population surveys as well as for clinical and research purposes. The CIDI is available in lifetime and 12-month versions, and in both paper-and-pencil (human interview) and computer-administered forms. The latter version is suitable for self-administration (the CIDI-Auto) in cooperative non-psychotic subjects for evaluating anxiety and depression disorders [2]. Recently a CIDI short form of the interview (the CIDI-SF) was developed [3]. The CIDI-SF is available in a 12-month prevalence format, and evaluates seven DSM-IV mental disorders and two DSM-III R substance disorders: major depression, generalized anxiety, specific phobia, social phobia, agoraphobia, panic attack, obsessive-compulsive disorder, alcohol dependence, and drug dependence. Also for this version, training of the interviewer is required.

This paper is about the development of a questionnaire for assessing mental disorders, which originates from the CIDI-SF, in the framework of a research project promoted by the Italian National Institute of Health to investigate the relationship between psychiatric disorders and working condition among health care workers. The questionnaire called "Health Problems Questionnaire" (HPQ), was designed to be: self-administered, more comprehensive (covering all domains which were considered in the CIDI-SF, but also including some screening questions on eating and psychotic disorders), and provided with multilevel scales able to discriminate between individuals having current symptoms and those having symptoms in the past.

The objectives of this report are to describe the development, the main features and preliminary validation of the HPQ.

## 2. Methods

### 2.1. Preliminary version of the questionnaire

The 2 components of the original CIDI-SF, the respondent questionnaire and the interviewer evaluation guide [4,5], were combined in a single questionnaire that could be entirely self-administered by the respondent. Four questions for screening psychotic disorders an two questions for screening eating disorders were added (see later). To obtain an Italian version of the CIDI-SF, an initial translation was produced by a psychiatrist, author of this paper (PM), with previous experience in translating English instruments into Italian [6]. This first version was independently revised by a clinical psychologist (AG). All suggestions were discussed by the translator with the reviewer, and those deemed to be relevant were included in a second version.

### 2.2. Content and face validity of the preliminary version

We sent this preliminary version of the questionnaire to 2 psychologists and 1 psychiatrist working at the 'Roma D' Mental Health Department. These professionals were asked to comment on the wording of the questions, the self-administered feasibility, and the congruence with the general principles of the CIDI-SF. They were also asked about the usefulness of the questionnaire. All professionals felt that all questions are relevant and thought the questionnaire would be helpful. However, to yield a more accurate assessment of depressive symptoms they felt that questions on hypochondria, restlessness, sense of guilt, and hopelessness should have been added.

To assess face validity of the preliminary version, two focus groups consisting of 7 health workers (1 doctor, 2 psychologists, 2 nurses, 1 medical radiology technician, and 1 security guard) at the Department of Psychosomatics and Hospital Medicine of the 'Rome E' (Roma, Italy) Health Department and 8 health workers (4 doctors, 1 sociologist, 1 psychologist, 1 nurse and 1 social worker) at the General Hospital Teramo (Italy) 1 were held. We developed a guide for the focus groups facilitators which came from the methodology described by Kitzinger [7]. The facilitator had a series of open ended questions to encourage participants not to approve in a indiscriminate way but to identify deficiencies on the clarity and relevance of the questionnaire and layout.

From the majority of participants the instrument covered mental health problems in a comprehensive way and all commented that questions were helpful for better understanding or treating problems that may affect employees and for planning workplace interventions. Some workers commented that questions on drug dependence were irrelevant for the majority of Italian health care workers.

### 2.3. Intermediate version of the questionnaire

On the basis of the content and face validity procedures, some depressive symptoms questions were added, and the number of questions on drug dependence was reduced (see later).

### 2.4. Test-retest reliability

Forty-five health professionals with different roles participated in a test-retest reliability (T1–T2: 2 weeks) of the intermediate version of the questionnaire. Reasons for differences in the answers between the first and second completion were investigated to evaluate if they were due to a real change of opinion or to ambiguity of the questions and response categories. Test-retest reliability was measured for each of the main questions (items) of the questionnaire (see Table 1). After the reliability study, slight changes were made.

**Table 1:** Reliability of the 29 initial probe items of the HPQ (N = 45).

Content Item	Weighted Cohen's kappa
1. Thinking to have a severe physical disease	0.75
2. Restlessness	0.64
3. Feeling tired	0.54
4. Trouble with sleep	0.62
5. Trouble concentrating	0.73
6. Feeling worthless	0.59
7. Thoughts about death	0.76
8. Sense of guilt	0.58
9. Hopelessness	0.64
10. Change in weight (lost weight)	0.84
11. Change in weight (gained weight)	0.93
12. Feeling sad, blue, or depressed	0.78
13. Loss of interest	0.56
14. Being too thin and to eat too little or do too exercise to lose weight	0.79
15. Eating a large amount of food and then doing something to not gain weight	0.93
16. Feeling worried, tense, or anxious	0.64
17. Having a strong fear about specific things	0.77
18. Having a strong fear about doing things in front of other people	0.67
19. Having a strong fear about being in a crowd, away from home alone, etc.	0.58
20. All of a sudden feeling frightened, anxious, uneasy	0.60
21. Number of glass of wine, or tins of beer (average in any single day)	0.79
22. Number of shots of liquor, or bitter, etc. (average in any single day)	0.82
23. Using psychotropic drugs	0.74
24. Being excessively happy, high spirited, thrilled, self-confident	0.64
25. Doing something over and over again or repeating gestures in a same order	0.38
26. Having unpleasant, exaggerated, unreasonable thoughts	0.70
27. Hearing voices	1.00
28. Feeling that own thoughts are controlled by some outside force	0.95
29. Feeling that some people are plotting or harming	0.58

### 2.5. Final version of the questionnaire

The final version of the instrument included 29 main questions and 56 secondary questions. Like the original CIDI-SF, the entire questionnaire used a stem-branch logic in which initial diagnostic stem questions are used to skip-out people who are least likely to have a psychiatric disorder before they are asked further additional questions.

#### 2.5.1. Major Depression (MD)

In the CIDI-SF Major Depression (MD) section there are two ways to meet the diagnostic stem requirement: either to endorse questions about having two weeks in a row of dysphoric mood (CIDI-SF questions No. A1-A1a-A1b) or to endorse questions about having two weeks of anhedonia (A9-A9a-A9b), lasting most of day, nearly everyday. If the diagnostic stem requirement is achieved, two identical series of additional symptom questions are asked both for respondents who endorse the dysphoric mood stem series or the anhedonia stem series. These additional questions refer to 1) losing interest (A1c; A9a and A9b), 2) feeling tired (A1d; A9c), 3) change in weight (A2b; A10b), 4) trouble with sleep (A3a; A11a), 5) trouble concentrating (A4; A12), 6) feeling down (A5; A13), and 7) thoughts about death (A6; A14). A subject is considered likely to have the disorder if he/she endorses questions about having dysphoric mood and 3 questions or more about having these additional symptoms or if he/she endorses questions about having anhedonia and 2 questions or more about having additional symptoms except losing interest.

In the HPQ [see Additional file 1], some modifications in content and layout innovations were done:

- four questions about hypochondria, restlessness, sense of guilt, and hopelessness were added;

- the response categories of the questions about having dysphoric mood and anhedonia, that in the CIDI-SF are dichotomous (yes/no), have been modified to evaluate also current and lifetime prevalence rates and sub threshold disorders (MD symptoms with less duration and frequency);

- the questions about how much longer respondents have had dysphoric mood and/or anhedonia were inserted into a box placed just below the stem questions (questions No. 12 e 13). Instructions are made to go into this box either respondents have felt sad, blue or depressed and/or they have had lost interest on most things in the past month or in the past year. A slight modification was made in the response categories for dysphoric mood/anhedonia persistence that in original CIDI-SF were *'every day' 'almost every day' 'less often'*. In this box, the individuals are also asked to report how many of the listed additional symptoms they have had during the 2 week period when feelings were worst;

- one question about difficulty to control over the symptoms (in the CIDI-SF this topic is assessed only for the Generalized Anxiety Disorder) was added;

- the two CIDI-SF questions evaluating contact with a doctor and contact with any other professionals were combined into a question that was inserted into the box together with the questions for evaluating use of medication, drugs or alcohol, and interference with daily functioning. Here and in any other relevant sections of the questionnaire, respondents are also asked about the amount of distress that could be caused by mental problems.

#### 2.5.2. Generalized Anxiety Disorder (GAD)

In the CIDI-SF the diagnostic stem requirement for GAD is met if the respondent reports a period of feeling worried, tense, or anxious (B1 or B1a = 1) that lasted at least 6 months (B2a or B2b > 6 months). If this stem requirement is achieved, further qualifiers are asked to determine whether the anxious period was stronger than in other people (B4 = 1), lasted more days than not (B5 = 1), and involved worrying about more than one thing (B6 = 2 or B8 = 1). The difficulty to control over the worries is then assessed by means of three questions (B7, B9–B10). Seven physiological symptoms (B12a-g), that may characterize the worried period, are finally assessed and the probability of having the disorder assigned if at least 3 of these symptoms are endorsed.

In the HPQ the following modifications in content and layout innovations were done:

- to obtain the same requirement in content but shortened in form, the stem questions were combined into the following question and responses: *'Was there ever a time when you felt worried, tense or anxious a lot more than most people would in your situation and about more than one thing at the same time? (response categories: Yes, recently, almost every day; Yes, in the past year, for at least 6 months, almost every day, but not at the present; Yes, for the same duration and frequency, but only before the past year; Yes, but with less duration and frequency in the past month; Yes, but with less duration and frequency in the past 12 months, not in the past month; Previously with less duration and frequency, or never);*

- because the criterion of difficulty to control is achieved even if only one of the three questions is endorsed, questions were reduced from 3 to 1 (*'When you had these feelings or problems, how often have you been able to take your mind off your feeling or problems? Response categories: 'never', ' rarely', ' more often');*

- the questions about physiological symptoms, identical to those reported by the CIDI-SF, were inserted into a box placed just below the stem question. Instructions were made to go into the box if the stem question was endorsed. In the same box, questions evaluating contact with an health care provider or other professionals, use of medication, and interference with daily functioning and distress were inserted.

#### 2.5.3. Specific Phobia (SpP)

In the CIDI-SF this section begins by assessing types of unreasonably strong fears organised into four categories: natural environment (C1a), situational (C1b), animal (C1c), and blood-injection-injury (C1d). For each category one question is given. Afterwards, two questions evaluating the frequency of anxious response to the stimulus (C3) and for how long the fears have been experienced (C4). Respondents are finally asked whether the fears interfered a lot with the activities of life (C5), or caused distress (C6), or were excessive or unreasonable (C7–C8).

In the HPQ:

- because diagnostic stem requirement for SpP is met if at least one type of fear is endorsed, questions were reduced from 4 to 1, and examples of fears of all categories were listed between brackets (i.e., *There are things that make some people excessively afraid (e.g. heights, storms, swimming pool, lake, or being in a closed space like a cave, tunnel, elevator, airplane, or animals like birds, rats, bugs, or seeing blood, getting a shot or injection). Did you have an unreasonably (much stronger than it should be), excessive (much stronger than in other people) fear and you get very upset or you avoid any of those things?*);

- response categories of the stem question were designed to allow to meet the frequency (at least most of the time) and duration (at least 3 months) CIDI requirements *(Yes, recently, most of the time; Yes, most of the time for at least three months, in the past 12 months, but not at present; Yes, for the same duration and with the same frequency, but only before the past year; Yes, but with less duration and frequency in the past month; Yes, but with less duration and frequency in the past 12 months, not in the past month; Previously with less duration and frequency, or never);*

- questions on excessive or unreasonable fear were omitted because incorporated in the above mentioned stem question. Question evaluating whether the phobia interfered with the respondent's life or caused distress was placed into a box just below the stem question. In the same box, a question evaluating contact with an health care provider or other helping professionals and a question evaluating use of medication, drugs or alcohol, that were not included in the CIDI-SF, were inserted. Instructions on going into the box if respondents had reported having experienced a specific phobia in the past month or in the past 12 months were given;

- one question about difficulty to control over the symptoms (identical to that used in the MD and GAD sections of the present questionnaire) was added.

#### 2.5.4. Social Phobia (SoP)

- As in the SpP section, the initial stem questions series (D1a-D1f) were reduced from 6 to 1, and examples of different social anxiety categories were listed between brackets: [*'Some people have an unreasonably strong fear when doing things in front of other people, (i.e. giving a speech or speaking in public or talking to people, or eating or drinking, or writing while someone watches, or taking part in a meeting or class, or going to a party). Did you have an unreasonably (much stronger than it should be), excessive (much stronger than in other people) fear and get very upset or avoid any of those situations?'*];

- response categories of the stem question were designed to allow to meet frequency (at least most of the time) and long duration (at least 3 months) CIDI requirements *(Yes, recently, most of the time; Yes, most of the time for at least three months, in the past 12 months but not at present; Yes, most of the time, for at least three months, before the last year; Yes, but with less duration and frequency in the past month; Yes, but with less duration and frequency in the past 12 months, not in the past month; Previously with less duration and frequency, or never);*

- in the CIDI-SF, if previous requirements were reached, respondents were finally classified as having the disorder based on whether they also have reported to have phobia that interfered a lot with the activities of life (D5), or that caused distress (D6), or that were excessive or unreasonable (D7–D8). In the PHQ, questions on excessive or unreasonable social phobia were omitted because incorporated in the above cited stem question. A question evaluating whether the social phobia interfered a lot with the respondent's life or caused distress was placed into a box placed just below. As in the SpP section, questions evaluating contact with an health care provider or other helping professionals, use of medication, drugs or alcohol, not included in the CIDI-SF, were added;

- the question about difficulty to control over the symptoms, identical to that used in the previous sections, was added.

#### 2.5.5. Agoraphobia (AGO)

- As in the SpP and SoP sections, the initial stem questions series (E1a-E1e) was reduced from 5 to 1 question, and different agoraphobia categories were then listed ('*There are situations in which most people don't have any problems, instead other people have a strong fear, i.e. being in a crowd or standing in line, or being away from home alone, or travelling alone in a bus, train, or car, or being in a public place like a department store. Do you have an unreasonably strong fear or avoid these types of situations because you are afraid that you might faint, lose control, or you worry that you might be trapped without any way to escape, or that help might not be available if you needed it?*);

- response categories of this question were designed to meet requirements for CIDI-SF AGO diagnosis in terms of anxious response frequency (at least most of the time) and duration (at least 3 months). The subsequent CIDI-SF questions for evaluating the characteristic symptoms of agoraphobia (i.e. fear of fainting or loosing control, fear of being trapped without escape, and fear that help might be available if it is needed) were omitted because incorporated in the above mentioned question and responses categories;

- a question evaluating whether the agoraphobia interfered (or caused distress) with the respondent's life or activities was placed into a box just below the stem question. In the same box, questions evaluating contact with an health care provider, use of medication, drugs or alcohol, not included in the CIDI-SF, were inserted;

- the question about difficulty to control over the symptoms, identical to that used in the previous sections, was added.

#### 2.5.6. Panic attack (PA)

- In the CIDI-SF, this section begins by assessing whether a panic attack has occurred in the past 12 months. Afterwards, six symptom questions are asked about pounding heart, discomfort in the chest, sweating, trembling, hot flashes or chills, and sense of unrealness. Respondents can be classified as having the disorder if they have at least three of these symptoms. In our questionnaire all these questions are combined as follows: *'Did you ever have a panic attack or an anxiety crisis when all of a sudden and without reason you felt very uneasy, anxious, and your heart pounded or raced, or you trembled or shake, or you had tightness in your chest, or you felt to faint, or things around you seemed unreal?'*

- as done in the other sections of the HPQ, the original dichotomous (yes/no) response categories of the stem question were modified to assess also 1-month, and lifetime prevalence rates (i.e., *Yes, in the past month; Yes in the past 12 months, but not in the past month; Only before the past 12 months; Never*);

- CIDI-SF questions that evaluate exclusions for attacks that occurred as a result of being in a life-threatening situation (F1b), being in a danger or at the centre of attention (F4), or being in a situation that provokes unreasonably strong fears (F5a) were omitted;

- the question evaluating whether the problem interferes with the respondent's life or activities, or causes distress, and questions evaluating contact with an health care provider or other helping professionals, use of medication, drugs or alcohol, not included in the CIDI-SF, were added and placed into a box just below the question on the panic attack occurrence;

- the question about difficulty to control over the symptoms, identical to that used in the previous sections, was added.

#### 2.5.7. Alcohol dependence (AD)

- In the CIDI-SF, a stem question assesses what is the largest number of drinks the respondent has had in any single day during the past 12 months (G1). The interviewer specifies that 'drink' is either a bottle of beer, a wine cooler, a glass of wine, a shot of liquor, or a mixed drink. In the HPQ, two questions were given on this topic: *'On average, how many glass of wine, or glass or tins of beer you had in a single day during the past month? *and *'On average, how many shots of liquor, or bitter, or alcoholic appetizer, or coffees with a dash of brandy you had in a single day during the past month?' *Note that the reference time period was the month preceding the interview, not the previous year, as in the CIDI-SF, and that '*the largest number*' was replaced by the average number of drinks because *'the largest number' *of drinks could occur just once, in this case it could not likely to be related with abuse;

- CIDI-SF questions to assess symptoms of DSM-III-R alcohol dependence were included into a box just below the initial question. The question on strong desire or urge to drink *('During the past 12 months, did you have such a strong desire or urge to drink that you could not keep from drinking?') *was removed because considered redundant. It was replaced by a question derived from the CAGE questionnaire (i.e. *'During the past 12 months, have you tried to stop or to cut down your drinking but with no success?'*) considered more adequate to assess alcohol dependence.

#### 2.5.8. Eating disorders (ED)

This section, as previously said, is not covered by the CIDI-SF, thus is completely innovative. Two questions about problems subjects might have had either with eating or weight introduce this section [*'Did you ever a time in your life when many people tell you that you was too thin and that you ate too little or that you took too exercise to lose more weight without needing that you lost weight?; Did you ever a time in your life when you ate a large amount of food (e.g. the whole of refrigerator food) until you felt sick, and after you made yourself vomit or other things in order to control your weight?']*. Response categories were developed to assess 1-month, 12 months and lifetime prevalence rates (i.e., *Yes, in the past month; Yes in the past 12 months, but not in the past month; Only before the past 12 months; Never*).

Three additional questions about things done in order to control weight *(In the last 12 months, in order to control your weight: Did you take drugs or pills? Did you make yourself vomit? Did you take laxatives or enemas?)*, and two questions about whether the problems have caused distress or interference, and about contact with a doctor or a dietician, respectively, were inserted into a box that was placed just below the two introductive questions.

#### 2.5.9. Drug dependence

This section, was limited to only one question, about taking psychotropic drugs without a doctor' prescription or in larger amount than prescribed, during the past 12 months, followed by two additional questions about interference with life and trying to stop.

#### 2.5.10. Obsessive disorder

- Because the obsessive requirement of the CIDI-SF was achieved either the question on the persistent idea that hands are dirty (OCD1) or the question on the persistent idea that the respondent might harm someone (OCD2) are endorsed, in the HPQ these questions were reduced to one including both kind of ideas (i.e., *'Have you ever had certain unpleasant thoughts, exaggerated, recognised as unreasonable, and against your human values (e.g. about harming yourself or other persons, swearing, or having a strong fear of dirt) over and over again that kept entering your mind against your wishes?'*);

- the response categories were identical to those used for panic attack assessment;

- the question about whether the obsessions are recognized as unreasonable (OCD3) was omitted because incorporated in the initial question;

- contact with other than doctor health care providers or helping professionals, and use of medication, drugs or alcohol, not included in the CIDI-SF, was evaluated together with contact with a doctor and placed into a box just below;

- response categories of questions assessing if obsessions have caused marked distress or interference (OCD6) were modified from *yes/no *to *a lot, somewhat, a little, not at all*.

#### 2.5.11. Compulsive disorders

- The CIDI-SF questions to assess any compulsions such as repeatedly checking that a door is locked or performing activities according to rigid rules (OCD7–9) were combined into only one question (i.e. '*Have you ever had to do something over and over again even though you know it is foolish, or unnecessary or overdone but you can't resist doing it – things like washing your hands again and again or going back several times to be sure you have locked a door or turned off the stove, or counting the squares in a tile floor, or repeating the same gestures in a same certain order or saying certain words over and over, either aloud or to yourself?');*

- the response categories were identical to those used for obsessions assessment;

- the question about whether the compulsions are recognized as unnecessary (OCD11) was omitted because incorporated in the stem question;

- questions evaluating contact with an health care provider or other helping professionals, use of medication, drugs or alcohol, not included in the CIDI-SF, were added and placed into a box just below the stem question;

- response categories of questions assessing if compulsions have caused marked distress or interference (OCD13) were modified from *yes/no *to *a lot, somewhat, a little, not at all*.

#### 2.5.12. Psychotic disorders

This section, not covered by the CIDI-SF, consists of five questions about hypomania, hearing hallucinations, thought insertion, paranoia derived from the Psychosis Screening Questionnaire[8], and delusions. Also for these questions, the response categories were realised to assess 1-month, 12-month and lifetime prevalence rates, and, like the others PHQ sections, include an additional box for evaluating contact with an health care provider or other helping professionals, use of medication, drugs or alcohol, and interference or distress as a result of these problems.

1. 'Did you ever have a period when you became so happy, high spirited, thrilled, self-confident that your relatives and friends worried about you or you did something that caused you to get into troubles?'

2. 'Did you ever hear voices talking to you when there was no one around or any place from which could come out?'

3. 'Have you ever felt that your thoughts were controlled by some outside force or person?'

4. 'Have you ever felt that there was a plotting against you or some people were acting to harm you or your interests?'

5. Have you ever experiences or beliefs that are so unusual that all or almost all other people would find very hard to believe?

### 2.6. Factor analysis and internal consistency

#### 2.6.1. Subjects

The study took place in a general hospital (total staff of about 700; catchment area of about 200,000), located in a mid-size urban area in central Italy, between February and July 2003. The hospital management asked all to participate, emphasising that anonymity and confidentiality would be assured. Sealed drop-off boxes were placed in each ward of the hospital. Staff were solicited to complete the questionnaire, but were told they were of course free not to do it or to avoid answering questions they did not like. A passive consent approach was adopted (i.e., the receipt of completed answers was taken to imply consent).

#### 2.6.2. Factor analysis

An exploratory factor analysis was conducted on the 29 initial probe items of the HPQ.

#### 2.6.3. Internal consistency

It has been determined on the entire questionnaire and on each of the factors identified.

#### 2.6.4. Statistical analysis

Test-retest reliability was measured by calculating the Cohen's weighted kappa [9]. The intra class correlation coefficient would have been also appropriate. However, because we used the quadratic weighting scheme, which bases disagreement weights on the square of the amount of discrepancy, the weighted kappa is exactly identical to the intra-class correlation coefficient [9].

Principal component analysis was conducted and the Kaiser criteria [10] were used to determine the number of factors of the questionnaire. Cronbach' alpha coefficient was used to evaluate internal consistency.

All statistical analyses were carried out using SPSS 15.0 for Windows.

## 3. Results

### 3.1. Test-retest reliability

Table 1 summarise the reliability of the 29 main items of the HPQ. Overall, the agreement between the first and the second set of results was good. For 6 items (21%), the weighted kappa was higher than 0.8 and for 15 items (52%) it ranged from 0.6 to 0.8. For the remaining 8 items (27%) the weighted kappa approached 0.6, with exception of the item on compulsive behaviours (kappa = 0.34). This item was slightly modified adding 'more than 3 times', between brackets, after the sentence 'Have you ever had to do something over and over again ..'.

### 3.2. Factor analysis and internal consistency

#### 3.2.1. Subjects response rate

Of the 726 staff members, 537 (74%) returned the questionnaire; 23 questionnaires were discarded because they were lacking more than 10% of the data. Thus 514 questionnaires were considered in the analysis (response rate of 71%). Of the responders, 284 (67%) were females. Regarding the type of profession, 229 (45%) were nurses, 113 (22%) doctors, 63 (12%) auxiliary staff, 37 (7%) medical technicians, 21 (4%) belonged to other professions (biologists, chemists, midwives, social workers, dieticians, physical therapists, and speech pathologists), and 10 (2%) were administrative personnel (missing datum 8%). As regards age distribution, 82 (16%) were aged 26–34 years, 190 (37%) were aged 35–44 years, and 216 (42%) were aged 45 years or older (missing datum 5%). Responders and non-responders did not significantly differ in terms of demographic characteristics or type of profession.

#### 3.2.2. Factor analysis

Factor analysis resulted in seven components with an eigenvalue greater than 1.0. Together they explained 57.3% of the variance. Following varimax rotation, however, one of the factors appeared to be not interpretable to the content of the items; the analysis was repeated with a forced six-factor solution. This solution explained 53.6% of the variance.

All items load 0.40 or more on at least one factor, which the majority of the items loading 0.50 or more. Some items load on more than one factor, and in this case, in this phase, only the highest factor loading was considered. A first factor contained 12 items covering depressive (10 items), hypochondria (1 item), and generalised anxiety (1 item) symptoms. A second consisted of 4 items on obsessive disorder (1 items), thoughts about death (1 item), thought insertion (1 item) and paranoia (1 item). A third factor contained 3 items on psychotropic drug use (1 item), hypomania (1 item), and hearing hallucinations (1 item). A fourth factor included 4 items concerning all kind of phobias (3 items) and panic attack (1 item). A fifth factor consisted of 3 items covering anorexia and bulimia symptoms (2 items) and gaining weight caused by an excessive appetite (1 item). A sixth factor consisted of 2 items on alcohol abuse symptoms. The item 'Doing something over and over again or repeating gestures in a same order' correlated both with the factor 2 and 3.

#### 3.2.3. Internal consistency

The Cronbach's alpha for the entire questionnaire was 0.89. The Cronbach's alpha for factors from 1 to 6, was 0.87, 0.64, 0.60, 0.71, 0.49, and 0.67, respectively.

Because the Cronbach's alpha for the entire questionnaire was greater than within the factors, we also conducted a Pearson correlation analysis between each item and all others to investigate if the items within the identified factors should correlate more closely with one another. We found that findings were consistent with the previous factor analysis, except for the item 7 on *thoughts about death*, that was more closely correlated with the items of the first factor rather that with the items of the second factor, and the item 28 on *feeling that own thoughts are controlled by some outside force or person*, that was more closely correlated with the items of the third factor rather that with the items of the second factor. So, we have included the item 7 in the factor 1 and the item 28 in the factor 3, with the effect of improving the Cronbach's alpha for factors (0.89, 0.67, 0.71, 0.71, 0.49, and 0.67, respectively).

The factors were labelled: (1) depression, (2) obsessions-compulsions, (3) hypomania and psychotic symptoms, (4) phobia, (5) eating disorders, and (6) alcohol abuse (Table 2).

**Table 2:** Factor loading of the 29 initial probe items of the HPQ after varimax rotation (N = 514), for the following factors: f1 depression, f2 obsessions-compulsions, f3 hypomania and psychotic symptoms, f4 phobia, f5 eating disorders, f6 alcohol abuse.

Content Item	Factor loading					
	f1	F2	f3	f4	f5	f6

1. Thinking to have a severe physical disease	**0.40**	0.08	0.21	0.13	0.10	0.04
2. Restlessness	**0.46**	0.30	0.01	0.10	0.17	0.09
3. Feeling tired	**0.76**	0.02	0.12	0.07	0.10	0.15
4. Trouble with sleep	**0.59**	0.02	0.22	0.09	0.27	0.15
5. Trouble concentrating	**0.73**	0.06	0.29	0.11	0.08	0.08
6. Feeling worthless	**0.65**	0.37	0.02	0.19	0.03	0.14
7. Thoughts about death	**0.38**	0,66	0.11	0.01	0.03	0.08
8. Sense of guilt	**0.64**	0.20	0.14	0.11	0.09	0.07
9. Hopelessness	**0.57**	0.33	0.11	0.13	0.01	0.15
10. Change in weight (lost weight)	0.16	0.18	0.00	0.06	**0.55**	0.03
11. Change in weight (gained weight)	**0.42**	0.09	0.30	0.15	0.23	0.04
12. Feeling sad, blue, or depressed	**0.73**	0.15	0.05	0.14	0.13	0.04
13. Loss of interest	**0.71**	0.12	0.13	0.19	0.22	0.07
14. Being too thin and to eat too little or do too exercise to lose weight	0.30	0.10	0.07	0.05	**0.71**	0.04
15. Eating a large amount of food and then doing something to not gain weight	0.00	0.12	0.19	0.25	**0.61**	0.17
16. Feeling worried, tense, or anxious	**0.66**	0.25	0.05	0.24	0.33	0.12
17. Having a strong fear about specific things	0.19	0.08	0.06	**0.60**	0.32	0.03
18. Having a strong fear about doing things in front of other people	0.34	0.16	0.00	**0.61**	0.00	0.03
19. Having a strong fear about being in a crowd, away from home alone, etc.	0.13	0.12	0.27	**0.69**	0.07	0.12
20. All of a sudden feeling frightened, anxious, uneasy	0.23	0.44	0.01	**0.55**	0.15	0.01
21. Number of glass of wine, or tins of beer (average in any single day)	0.05	0.14	0.11	0.00	0.03	**0.82**
22. Number of shots of liquor, or bitter, etc. (average in any single day)	0.05	0.02	0.09	0.10	0.12	**0.82**
23. Using psychotropic drugs	0.07	0.04	**0.53**	0.36	0.10	0.14
24. Being excessively happy, high spirited, thrilled, self-confident	0.19	0.29	**0.75**	0.04	0.09	0.09
25. Doing something over and over again or repeating gestures in a same order	0.16	**0.39**	*0.38*	0.13	0.24	0.14
26. Having unpleasant, exaggerated, unreasonable thoughts	0.26	**0.58**	0.22	0.09	0.20	0.02
27. Hearing voices	0.03	0.27	**0.72**	0.07	0.05	0.10
28. Feeling that own thoughts are controlled by some outside force	0.05	0.71	**0.26**	0.17	0.05	0.02
29. Feeling that some people are plotting or harming	0.08	**0.47**	0.03	0.22	0.19	0.28

## 4. Discussion

We developed and evaluated a self-administered questionnaire for assessing the major mental disorders with particular emphasis placed on feasibility and reliability.

The questionnaire was based on the CIDI instrument. While several studies show adequate reliability and validity for the CIDI in clinical samples, a criticism is that its reliability and validity in general population samples needs to be still established [11]. It is worth underlying that our questionnaire was designed for and is being used in a non-clinical sample.

The advantages to be a self-filled instrument include that it does not require skilled interviewers, and is free from interviewer bias. The need to be cautious about generalising results from diagnostic interviews delivered by an interviewer to self administered interviews was already highlighted by a recent comparison of the CIDI self-administered (computerised) with the standard administration [12]. It is concluded that the agreement between the two formats was acceptable, at least as anxiety and depressive disorders are concerned.

Face and content validity and reliability of our questionnaire have been assessed. These were deemed to be acceptable by the three mental health professionals and the two focus groups who evaluated the preliminary version of the questionnaire. The level of test-retest reliability was quite high, as shown by the Cohen's weighted kappa, which ranged from 0.6 to 1.0 for the majority of the items.

The factor analysis and the subsequent Pearson inter item correlation analysis were performed in a fairly large sample, and the percentage of total variance explained by factors was quite high. Of the factors, five were consistent on depressive, phobic, eating, alcohol abuse, and obsessive-compulsive disorders. It could be argued that the item on *feeling that some people are plotting or harming *would be more consistent with the hypomania and psychotic symptoms factor that with the factor on obsessive-compulsive disorders in which it was included. This item has been already modified from *'Have you ever felt that there was a plotting against you or some people were acting to harm you or your interests?' *in *'Have you ever felt that some people were acting to harm you or your interests, perhaps in a plot against you?'*.

The high response rate and the few questionnaires not completed could provide some indications as to the questionnaire's acceptability. In fact, it should be reaffirmed that the questionnaires were anonymous and sealed drop-off boxes were placed in each ward of the hospital to insert the filled-in questionnaires. Hence, there was no possibility to identify the non respondents. The high response rate might be due to the main advantages of the questionnaire, i.e brevity and user-friendliness. It could be noted that, from the point of view of the acceptability, the CIDI-Auto was found to be less embarrassing but too long in comparison with the CIDI.

Despite its brevity, the questionnaire includes most important items on disorders not covered by the CIDI-SF from which it originated. Because of response categories used, which allow to evaluate current, 12 months, and lifetime prevalence rates, its use in longitudinal surveys might allow to easily assess change over time of mental disorders prevalence.

In the literature, some other self-report measures of mental disorder prevalence already exist, such as The Primary Care Evaluation of Mental Disorders (PRIME-MD), the Patient Health Questionnaire (PQH) [13] and The Inventory to Diagnose Depression (IDD) [14]. However, the IDD only cover the major depressive disorder. The PHQ is short, easily scored and has high specificity despite modest sensitivity [15]. It might be useful in setting where missing some cases of depression is not considered a major problem and a quick diagnosis is needed. Moreover, it should be noted that in the original validation study the PHQ was not fully self-administered; the general practitioner briefly reviewed the questionnaire with each patient and asked additional questions to clarify the answers.

Our instrument has some limitations. First, the reports about the experience in the whole life inquired by *before the past 12 months *could be subjected to recall bias [16]. Although we realize that the inclusion of this inquiry is controversial, one should keep in mind that it may be seen as a resource not only to estimate lifetime prevalence but also to investigate a subjective opinion that might have an influence on the future response to stress for events and situations.

Second, the process of validation of the HPQ is not complete and needs to be supplemented by a further validation to estimate its concurrent validity. Future evaluations could also include the analysis of discriminant validity and sensitivity to change.

Third, we studied only health care workers. We have done so because, as already said, the instrument was developed in the framework of a project focused on health care workers. Hence, our findings might not generalize to other populations. Some differences with the general population might exist because of the higher average level of education of our sample.

Finally, the items of the instrument which were entirely derived from the CIDI-SF were not formally back-translated. They have been translated into Italian and then for this presentation retranslated in English language. It is worth noting that they are very similar to the original expressions of the CIDI-SF.

## Authors' contributions

AG and PM participated in the sequence alignment and drafted the manuscript. PM conceived of the study. AG participated in its design and coordination and performed the statistical analysis. All authors read and approved the final manuscript.

## Supplementary Material

Additional file 1 The two initial pages of the Health Problems Questionnaire (HPQ) (presentation in English and in Italian) The text provided represent the two initial pages of the Health Problems Questionnaire (HPQ) both in English and in Italian language. Click here for file
