# Supplementary material for: Development, reliability and factor analysis of a self-administered questionnaire which originates from the World Health Organization's Composite International Diagnostic Interview – Short Form (CIDI-SF) for assessing mental disorders
Source: Clin Pract Epidemiol Ment Health. 2008 Apr 10;4:8. doi: 10.1186/1745-0179-4-8 (PMC2329624; doi:10.1186/1745-0179-4-8)
Supplement: Additional file 1 — The two initial pages of the Health Problems Questionnaire (HPQ) (presentation in English and in Italian) The text provided represent the two initial pages of the Health Problems Questionnaire (HPQ) both in English and in Italian language. [file 1745-0179-4-8-S1.doc]

**The Health Problems Questionnaire**

Istituto Superiore di Sanità – Roma (Italy)

*Derived from the WHO CIDI - SF , 1998*

*We would like to know something about health problems that you may have. Your answers will be useful to us for better understanding problems that may affect employees and for planning workplace interventions. For the following questions, please, place an “x” in the circle next to the answer that best correspond to your condition. We ask that you read the questions as carefully as possible. If you make an error, write “NO” next to the incorrect answer and place another “x” in the correct circle. We thank you in advance for your greatly appreciated collaboration.*

**a) Date of compilation** ____ ____ ____ **b) Gender** ➀ male ➁female

**c) Age:** ➀less than 25 years ➁25-34 ➂35-44 ➃45-54 ➄55 or more

**d) Profession**

➀doctor ➃auxiliary

➁nurse ➄other professions (biologist, chemist, midwife, social worker, dietician,

➂medical technician physical therapist, and speech pathologist)

➅administrative personnel

**1) Was there ever a time when you felt** **to have a severe disease of which doctors couldn’t find the cause**?

➀ Yes, for at least two weeks in a row, most of day, nearly everyday, in the past month

➁ Yes, for at least two weeks in a row, most of day, nearly everyday, in the past 12 months, but not in the past month

➂ Yes, for the same duration and with the same frequency but only before the past 12 months

➃ Yes, but with less duration and frequency, in the past month

➄ Yes, but with less duration and frequency in the past 12 months, not in the past month

➅Previously with less duration and frequency, or never

**2) Was there ever a time when you felt** **so restless that you had to be moving all the time** (e.g., you couldn’t sit still and paced up and down, or you couldn’t keep still your arms and legs when sitting)**?**

➀ Yes, for at least two weeks in a row, most of day, nearly everyday, in the past month

➁ Yes, for at least two weeks in a row, most of day, nearly everyday, in the past 12 months, but not in the past month

➂ Yes, for the same duration and with the same frequency but only before the past 12 months

➃ Yes, but with less duration and frequency, in the past month

➄ Yes, but with less duration and frequency in the past 12 months, not in the past month

➅Previously with less duration and frequency, or never

**3) Was there ever a time when you felt** **lack on energy or more tired than is usual for you, even if you has not been working very hard**?

➀ Yes, for at least two weeks in a row, most of day, nearly everyday, in the past month

➁ Yes, for at least two weeks in a row, most of day, nearly everyday, in the past 12 months, but not in the past month

➂ Yes, for the same duration and with the same frequency but only before the past 12 months

➃ Yes, but with less duration and frequency, in the past month

➄ Yes, but with less duration and frequency in the past 12 months, not in the past month

➅Previously with less duration and frequency, or never

**4**) **Was there ever a time when you had trouble sleeping?**

➀ Yes, for at least two weeks in a row, most of night, nearly everyday, in the past month

➁ Yes, for at least two weeks in a row, most of night, nearly everyday, in the past 12 months, but not in the past month

➂ Yes, for the same duration and with the same frequency but only before the past 12 months

➃ Yes, but with less duration and frequency, in the past month

➄ Yes, but with less duration and frequency in the past 12 months, not in the past month

➅Previously with less duration and frequency, or never

**5**) **Was there ever a time when you had trouble concentrating** **on something that you did, e.g. in working or reading the newspaper or watching TV programs that you liked?**

➀ Yes, for at least two weeks in a row, most of day, nearly everyday, in the past month

➁ Yes, for at least two weeks in a row, most of day, nearly everyday, in the past 12 months, but not in the past month

➂ Yes, for the same duration and with the same frequency but only before the past 12 months

➃ Yes, but with less duration and frequency, in the past month

➄ Yes, but with less duration and frequency in the past 12 months, not in the past month

➅Previously with less duration and frequency, or never

**6**) **Was there ever a time when you felt** **down on yourself, on your abilities, or worthless?**

➀ Yes, for at least two weeks in a row, most of day, nearly everyday, in the past month

➁ Yes, for at least two weeks in a row, most of day, nearly everyday, in the past 12 months, but not in the past month

➂ Yes, for the same duration and with the same frequency but only before the past 12 months

➃ Yes, but with less duration and frequency, in the past month

➄ Yes, but with less duration and frequency in the past 12 months, not in the past month

➅Previously with less duration and frequency, or never

**7**) **Was there ever a time when you felt** **that your life was worthless?**

➀ Yes, for at least two weeks in a row, most of day, nearly everyday, in the past month

➁ Yes, for at least two weeks in a row, most of day, nearly everyday, in the past 12 months, but not in the past month

➂ Yes, for the same duration and with the same frequency but only before the past 12 months

➃ Yes, but with less duration and frequency, in the past month

➄ Yes, but with less duration and frequency in the past 12 months, not in the past month

➅Previously with less duration and frequency, or never

**8) Was there ever a time when you felt** **guilty or remorse for something you have done?**

➀ Yes, for at least two weeks in a row, most of day, nearly everyday, in the past month

➁ Yes, for at least two weeks in a row, most of day, nearly everyday, in the past 12 months, but not in the past month

➂ Yes, for the same duration and with the same frequency but only before the past 12 months

➃ Yes, but with less duration and frequency, in the past month

➄ Yes, but with less duration and frequency in the past 12 months, not in the past month

➅Previously with less duration and frequency, or never

**9) Was there ever a time when you felt** **hopelessness, with no future?**

➀ Yes, for at least two weeks in a row, most of day, nearly everyday, in the past month

➁ Yes, for at least two weeks in a row, most of day, nearly everyday, in the past 12 months, but not in the past month

➂ Yes, for the same duration and with the same frequency but only before the past 12 months

➃ Yes, but with less duration and frequency, in the past month

➄ Yes, but with less duration and frequency in the past 12 months, not in the past month

➅Previously with less duration and frequency, or never

**10) Was there ever a time when you lost weight without trying as much as about 5 kilos in a short time?**

➀ Yes, in the past month

➁ Yes, in the past 12 months but not in the past month

➂ Before the past 12 months

➃ Previously or never

**11) Was there ever a time when you gained weight without trying as much as about 5 kilos in a short time?**

➀ Yes, in the past month

➁ Yes, in the past 12 months but not in the past month

➂ Before the past 12 months

➃ Previously or never

**12**) **Was there ever a time when you felt sad, blue or depressed for two week or more in a row?**

➀ Yes, for at least two weeks in a row, most of day, nearly everyday, in the past month

➁ Yes, for at least two weeks in a row, most of day, nearly everyday, in the past 12 months, but not in the past month

➂ Yes, for the same duration and with the same frequency but only before the past 12 months

➃ Yes, but with less duration and frequency, in the past month

➄ Yes, but with less duration and frequency in the past 12 months, not in the past month

➅Previously with less duration and frequency, or never

**13**) **Was there ever a time when you lost interest in most things that usually gave you pleasure like hobbies, work, cooking, gardening or playing with children?**

➀ Yes, for at least two weeks in a row, most of day, nearly everyday, in the past month

➁ Yes, for at least two weeks in a row, most of day, nearly everyday, in the past 12 months, but not in the past month

➂ Yes, for the same duration and with the same frequency but only before the past 12 months

➃ Yes, but with less duration and frequency, in the past month

➄ Yes, but with less duration and frequency in the past 12 months, not in the past month

➅Previously with less duration and frequency, or never

*If you felt sad, blue or depressed, or lost interest on most things - that is - if you placed an X in the circle* ➀ *or* ➁ *of the question #*12 *and/or of the question #*13*, GO into the box down here****🡻****.* *If not, JUMP directly to question #14, after the box***🡺***.*

**🡻13a) Please think of the two-week period when these feelings were worst. For how many days did you feel sad, blue, or depressed?**

➀ every day

➁ more than half of the days

➂ less often

**13b) During the day, how long did the feelings last on average?**

➀ all day long ➂ less than half

➁ most of the day ➃ only sometimes

**13c) During those two-weeks, DID YOU HAVE ALSO other feelings or problems enquired above, like: being tired, having trouble with sleep, having change in weight, having trouble concentrating, feeling worthless, or feeling that life wasn’t worth living?**

➀ yes, 3 or more of these problems

➁ yes, 2 of these problems

➂ only one

➃ none

**13d)** **When you had these feelings or problems, how often you has been able to take your mind off your feeling or problems?**

➀ never

➁ rarely

➂ more often

**13e) How much did these problems interfere with your life or activities, or cause marked distress?**

➀ a lot ➂ a little

➁ somewhat ➃ not at all

**13f) In the last 12 months, did you tell a doctor or any other helping professionals** (such as a psychologist, social worker, counsellor, nurse clergy, or other helping professionals such as an healing) **about these problems?**

➀ yes ➁ no

**13g) In the last 12 months, did you take medication or use drugs or alcohol more than once for these problems?**

➀ yes ➁ no

**🡺**

**Questionario sui problemi di salute**

Istituto Superiore di Sanità – Roma (Italy)

*Derivato da WHO CIDI - SF , 1998*

*Le seguenti domande riguardano le sue condizioni di salute. L a preghiamo di rispondere, facendo un segno sul cerchio vicino alla risposta che più corrisponde a come si è sentito o a cosa ha pensato nel periodo di riferimento. Se sbaglia, scriva NO accanto al segno fatto per errore e poi faccia il segno al posto giusto.*

**a) Data di compilazione** ____ ____ ____ **b) Sesso** ➀ maschio ➁femmina

**c) Età:** ➀meno di 25 anni ➁25-34 ➂35-44 ➃45-54 ➄55 o più

**d) Qualifica**

➀dirigente medico ➃OSA e ausiliario

➁ caposala e altro infermiere professionale ➄altre professioni (biologo, chimico, ostetrica, assistente sociale, dietista,

➂ tecnico sanitario (di laboratorio, terapista della riabilitazione, logopedista)

di radiologia, ecc.) ➅personale amministrativo

**1) Le è mai capitato di pensare di avere una malattia fisica grave di cui i medici non sono riusciti a trovare la causa?**

➀ Sì, per un periodo di almeno 2 settimane di fila, quasi tutti i giorni, per la maggior parte della giornata, negli ultimi 30 giorni

➁ Per almeno 2 settimane di fila, quasi tutti i giorni, per la maggior parte della giornata, nell'ultimo anno ma non negli ultimi 30 giorni

➂ Per la stessa durata e frequenza, ma solo prima dell’ultimo anno

➃ Per meno di 2 settimane, negli ultimi 30 giorni

➄ Per meno di 2 settimane, nell'ultimo anno ma non negli ultimi 30 giorni

➅Per meno tempo, prima dell’ultimo anno, oppure mai

**2) E’ mai stato tanto irrequieto da non riuscire a stare fermo** (ad esempio così irrequieto da continuare a muovere braccia e gambe stando seduto, o da non riuscire a star seduto e continuare a camminare avanti e indietro)**?**

➀ Sì, per un periodo di almeno 2 settimane di fila, quasi tutti i giorni, per la maggior parte della giornata, negli ultimi 30 giorni

➁ Per almeno 2 settimane di fila, quasi tutti i giorni, per la maggior parte della giornata, nell'ultimo anno ma non negli ultimi 30 giorni

➂ Per la stessa durata e frequenza, ma solo prima dell’ultimo anno

➃ Per meno di 2 settimane, negli ultimi 30 giorni

➄ Per meno di 2 settimane, nell'ultimo anno ma non negli ultimi 30 giorni

➅Per meno tempo, prima dell’ultimo anno, oppure mai

**3) Le è capitato di sentirti per diversi giorni di fila senza energie o stanco anche se non aveva lavorato molto?**

➀ Sì, per un periodo di almeno 2 settimane di fila, quasi tutti i giorni, per la maggior parte della giornata, negli ultimi 30 giorni

➁ Per almeno 2 settimane di fila, quasi tutti i giorni, per la maggior parte della giornata, nell'ultimo anno ma non negli ultimi 30 giorni

➂ Per la stessa durata e frequenza, ma solo prima dell’ultimo anno

➃ Per meno di 2 settimane, negli ultimi 30 giorni

➄ Per meno di 2 settimane, nell'ultimo anno ma non negli ultimi 30 giorni

**4**) **Ha mai avuto problemi di sonno?**

➀ Sì, per un periodo di almeno 2 settimane di fila, quasi tutti i giorni, per la maggior parte del tempo, negli ultimi 30 giorni

➁ Per almeno 2 settimane di fila, quasi tutti i giorni, per la maggior parte del tempo, nell'ultimo anno ma non negli ultimi 30 giorni

➂ Per la stessa durata e frequenza, ma solo prima dell’ultimo anno

➃ Per meno di 2 settimane, negli ultimi 30 giorni

➄ Per meno di 2 settimane, nell'ultimo anno ma non negli ultimi 30 giorni

➅Per meno tempo, prima dell’ultimo anno, oppure mai

**5**) **Ha mai avuto difficoltà nel concentrarsi nelle cose che faceva, ad esempio nel lavoro o nel leggere un giornale o nel seguire un programma televisivo che le piaceva?**

➀ Sì, per un periodo di almeno 2 settimane di fila, quasi tutti i giorni, per la maggior parte della giornata, negli ultimi 30 giorni

➁ Per almeno 2 settimane di fila, quasi tutti i giorni, per la maggior parte della giornata, nell'ultimo anno ma non negli ultimi 30 giorni

➂ Per la stessa durata e frequenza, ma solo prima dell’ultimo anno

➃ Per meno di 2 settimane, negli ultimi 30 giorni

➄ Per meno di 2 settimane, nell'ultimo anno ma non negli ultimi 30 giorni

➅Per meno tempo, prima dell’ultimo anno, oppure mai

**6**) **Le è mai successo di non avere fiducia in se stesso e nelle sue capacità o di sentirsi senza valore?**

➀ Sì, per un periodo di almeno 2 settimane di fila, quasi tutti i giorni, per la maggior parte della giornata, negli ultimi 30 giorni

➁ Per almeno 2 settimane di fila, quasi tutti i giorni, per la maggior parte della giornata, nell'ultimo anno ma non negli ultimi 30 giorni

➂ Per la stessa durata e frequenza, ma solo prima dell’ultimo anno

➃ Per meno di 2 settimane, negli ultimi 30 giorni

➄ Per meno di 2 settimane, nell'ultimo anno ma non negli ultimi 30 giorni

➅Per meno tempo, prima dell’ultimo anno, oppure mai

➅Per meno tempo, prima dell’ultimo anno, oppure mai

**7**) **Ha mai pensato che non valesse la pena di vivere?**

➀ Sì, per un periodo di almeno 2 settimane di fila, quasi tutti i giorni, per la maggior parte della giornata, negli ultimi 30 giorni

➁ Per almeno 2 settimane di fila, quasi tutti i giorni, per la maggior parte della giornata, nell'ultimo anno ma non negli ultimi 30 giorni

➂ Per la stessa durata e frequenza, ma solo prima dell’ultimo anno

➃ Per meno di 2 settimane, negli ultimi 30 giorni

➄ Per meno di 2 settimane, nell'ultimo anno ma non negli ultimi 30 giorni

➅Per meno tempo, prima dell’ultimo anno, oppure mai

**8) Le è mai capitato di sentirsi in colpa o di avere dei rimorsi?**

➀ Sì, per un periodo di almeno 2 settimane di fila, quasi tutti i giorni, per la maggior parte della giornata, negli ultimi 30 giorni

➁ Per almeno 2 settimane di fila, quasi tutti i giorni, per la maggior parte della giornata, nell'ultimo anno ma non negli ultimi 30 giorni

➂ Per la stessa durata e frequenza, ma solo prima dell’ultimo anno

➃ Per meno di 2 settimane, negli ultimi 30 giorni

➄ Per meno di 2 settimane, nell'ultimo anno ma non negli ultimi 30 giorni

➅Per meno tempo, prima dell’ultimo anno, oppure mai

**9) Ha mai pensato che non ci sarebbe stato più niente di bello e positivo nel suo futuro?**

➀ Sì, per un periodo di almeno 2 settimane di fila, quasi tutti i giorni, per la maggior parte della giornata, negli ultimi 30 giorni

➁ Per almeno 2 settimane di fila, quasi tutti i giorni, per la maggior parte della giornata, nell'ultimo anno ma non negli ultimi 30 giorni

➂ Per la stessa durata e frequenza, ma solo prima dell’ultimo anno

➃ Per meno di 2 settimane, negli ultimi 30 giorni

➄ Per meno di 2 settimane, nell'ultimo anno ma non negli ultimi 30 giorni

➅Per meno tempo, prima dell’ultimo anno, oppure mai

**10) Ha mai avuto problemi di mancanza di appetito per cui è dimagrito in poco tempo di 5 chili o più?**

➀ Sì, negli ultimi 30 giorni

➁ Sì, nell'ultimo anno ma non negli ultimi 30 giorni

➂ Solo prima dell’ultimo anno

➃ Prima dell’ultimo anno o mai

**11) Hai mai avuto problemi di eccesso di appetito per cui sei ingrassato in poco tempo di 5 chili o più?**

➀ Sì, negli ultimi 30 giorni

➁ Sì, nell'ultimo anno ma non negli ultimi 30 giorni

➂ Solo prima dell’ultimo anno

➃ Prima dell’ultimo anno o mai

**12**) **Si è mai sentito giù di morale, abbattuto, depresso?**

➀ Sì, per un periodo di almeno 2 settimane di fila, quasi tutti i giorni, per la maggior parte della giornata, negli ultimi 30 giorni

➁ Per almeno 2 settimane di fila, quasi tutti i giorni, per la maggior parte della giornata, nell'ultimo anno ma non negli ultimi 30 giorni

➂ Per la stessa durata e frequenza, ma solo prima dell’ultimo anno

➃ Per meno di 2 settimane, negli ultimi 30 giorni

➄ Per meno di 2 settimane, nell'ultimo anno ma non negli ultimi 30 giorni

➅Per meno tempo, prima dell’ultimo anno, oppure mai

**13**) **Ha mai perso interesse nel fare la maggior parte delle cose che di solito le piaceva fare, ad esempio il lavoro o i passatempi o anche semplicemente cucinare, curare i fiori, giocare con i bambini?**

➀ Sì, per un periodo di almeno 2 settimane di fila, quasi tutti i giorni, per la maggior parte della giornata, negli ultimi 30 giorni

➁ Per almeno 2 settimane di fila, quasi tutti i giorni, per la maggior parte della giornata, nell'ultimo anno ma non negli ultimi 30 giorni

➂ Per la stessa durata e frequenza, ma solo prima dell’ultimo anno

➃ Per meno di 2 settimane, negli ultimi 30 giorni

➄ Per meno di 2 settimane, nell'ultimo anno ma non negli ultimi 30 giorni

➅Per meno tempo, prima dell’ultimo anno, oppure mai

*Se si è sentito giù di morale, abbattuto, depresso o ha perso interesse per le cose -cioè –se ha messo un segno sul cerchio* ➀ *o* ➁ *alle domande 12 e/o 13, CONTINUI nel riquadro qui sotto****🡻****.* *Se no, SALTI direttamente alla domanda 14, dopo il riquadro***🡺***.*

**🡻13a) Pensi ora al periodo peggiore di 2 settimane o più. In quel periodo, per quanti giorni si è sentito giù di morale, depresso o senza interessi?**

➀ ogni giorno

➁ più della metà dei giorni

➂ per meno giorni

**13b) Nei giorni in cui era depresso o senza interessi, in media, quante ore al giorno si è sentito così?**

➀ tutto il giorno ➂ circa per metà giornata e comunque per almeno 3 ore

➁ la maggior parte del giorno ➃ per meno tempo

**13c) In quel periodo, ha avuto allo stesso tempo uno o più dei problemi di cui abbiamo parlato prima: irrequietezza, mancanza di energia, disturbi del sonno, perdita o aumento di peso, mancanza di concentrazione, sentirsi senza valore o senza fiducia in se stesso, pensare che non valesse la pena di vivere?**

➀ sì, tre o più dei problemi suddetti nello stesso periodo

➁ sì, due dei problemi suddetti

➂ solo uno

➃ nessuno

**13d)** **Quando ha avuto questi problemi, quanto è riuscito a distrarsi da questi problemi?**

➀ mai

➁ pochissime volte

➂ più spesso

**13e) Sempre negli ultimi 12 mesi, questi problemi le hanno procurato difficoltà e limitazioni nella sua vita e nelle sue attività o l’hanno fatta stare molto male?**

➀ moltissimo ➂ abbastanza

➁ molto ➃ poco o per niente

**13f) Negli ultimi 12 mesi ti sei rivolto a un medico o uno psicologo o anche a persone di cui si fida, come un prete o anche un guaritore, per questi problemi?**

➀ sì ➁ no

**13g) Negli ultimi 12 mesi ha preso più di una volta farmaci o alcol o droghe per questi problemi?**

➀ sì ➁ no
